# Silent Invader: The Enigmatic Case of Cardiac Angiosarcoma

**DOI:** 10.7759/cureus.101480

**Published:** 2026-01-13

**Authors:** FNU Arty, Devarashetty Shreya, Anoohya Vangala, Sankalp Acharya, Sai Rakshith Gaddameedi, Petro Vavrukh, Shazia M Shah

**Affiliations:** 1 Internal Medicine, Monmouth Medical Center, Long Branch, USA; 2 Internal Medicine, Rutgers Health/Monmouth Medical Center, Long Branch, USA; 3 Department of Pathology, Cooperman Barnabas Medical Center, Livingston, USA

**Keywords:** angiosarcoma, exertional dyspnea, primary cardiac tumor, recurrent pericardial effusion, recurrent pleural effusion

## Abstract

Cardiac angiosarcoma is a rare and aggressive malignant tumor that often masquerades as a more common cardiac condition. Patients frequently exhibit nonspecific symptoms, leading to delayed detection and a grim prognosis. Given the scarcity of documented cases, we present an intriguing case of primary cardiac epithelioid angiosarcoma with pleural metastasis, manifesting as recurrent pericardial and pleural effusions. This case highlights the diagnostic complexity of cardiac angiosarcoma, especially when typical right atrial masses are absent. Persistent, unexplained pericardial and pleural effusions should prompt consideration of malignancy, particularly when standard evaluations are inconclusive. Early tissue biopsy and a multidisciplinary approach are essential for the timely diagnosis and management of this rare but deadly condition.

## Introduction

Cardiac angiosarcoma is a rare and aggressive malignant tumor that often masquerades as a more common cardiac condition [1]. With its endothelial origin and epithelioid appearance, diagnosing this neoplasm presents significant challenges [2]. Patients frequently exhibit nonspecific symptoms, leading to delayed detection and a grim prognosis [3]. The cornerstone of treatment involves a multimodal approach encompassing radical surgery, radiation therapy, and chemotherapy [4]. Given the scarcity of documented cases, we present an intriguing case of primary cardiac epithelioid angiosarcoma with pleural metastasis, manifesting as recurrent pericardial and pleural effusions.

## Case presentation

A 56-year-old male with a past medical history of hypertension and hyperlipidemia presented to the emergency department with progressive exertional dyspnea and substernal chest discomfort. His clinical trajectory over the prior eight months had been marked by recurrent hospitalizations for idiopathic pericardial and pleural effusions, leading to multiple inconclusive diagnostic interventions. Despite pericardiocentesis, thoracenteses, and a comprehensive workup for infectious, malignant, and autoimmune etiologies, the underlying cause of his effusions remained elusive.

On arrival, the patient was afebrile and hemodynamically stable but exhibited tachypnea. Physical examination revealed distant heart sounds and decreased breath sounds at the right lung base, raising suspicion for both pericardial and pleural involvement. Laboratory analysis revealed elevated inflammatory markers, including the erythrocyte sedimentation rate (ESR) and C-reactive protein (CRP), alongside a coagulopathy and markedly elevated D-dimer levels. Serial troponins were negative (Table 1).

**Table 1:** Laboratory values mm/h: millimeters per hour, mg/L: milligrams per liter, FEU: fibrinogen equivalent units; ng/mL: nanograms per milliliter; s: seconds

Name of Test	Value	Normal Reference Value
ESR (Erythrocyte sedimentation rate)	32 mm/h	0-20 mm/h
CRP (C-reactive protein)	25.72 mg/L	< 7 mg/L
D-dimer	4.236 FEU	0.000-0.500 FEU
Troponin I	< 0.010 ng/mL	0.040-0.800 ng/mL
PT (Prothrombin time)	14.2 s	9.4-13.5 s
INR (International normalized ratio)	1.3	0.9-1.1 ratio
aPTT (Activated partial thromboplastin time)	37 s	24.6-37.8 s

**Figure 1:**
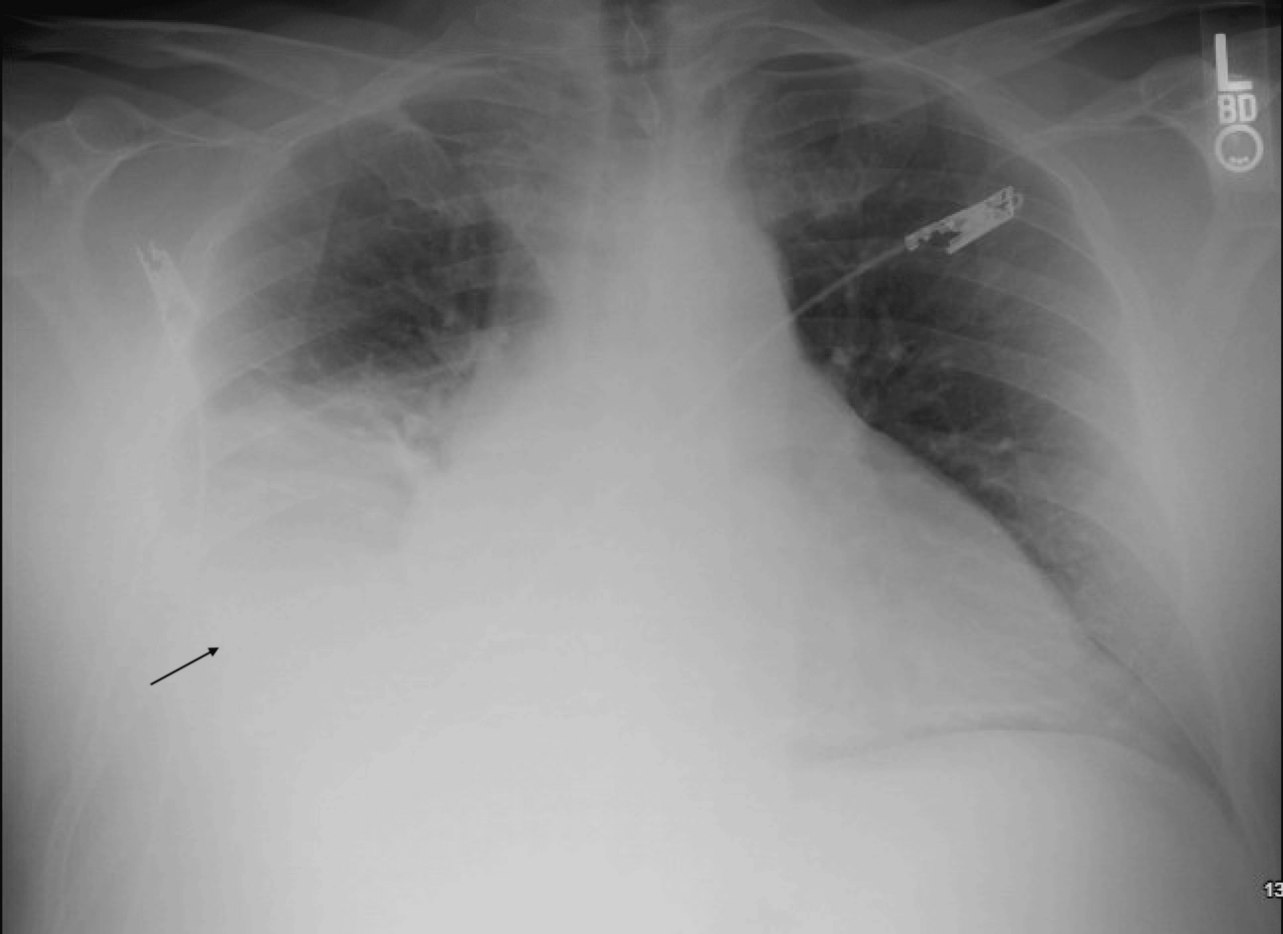
CXR (AP view): large right-sided pleural effusion (black arrow) CXR: Chest X-ray

Chest radiography (CXR) demonstrated a large right-sided pleural effusion with associated pulmonary vascular congestion (Figure 1).

**Figure 2:**
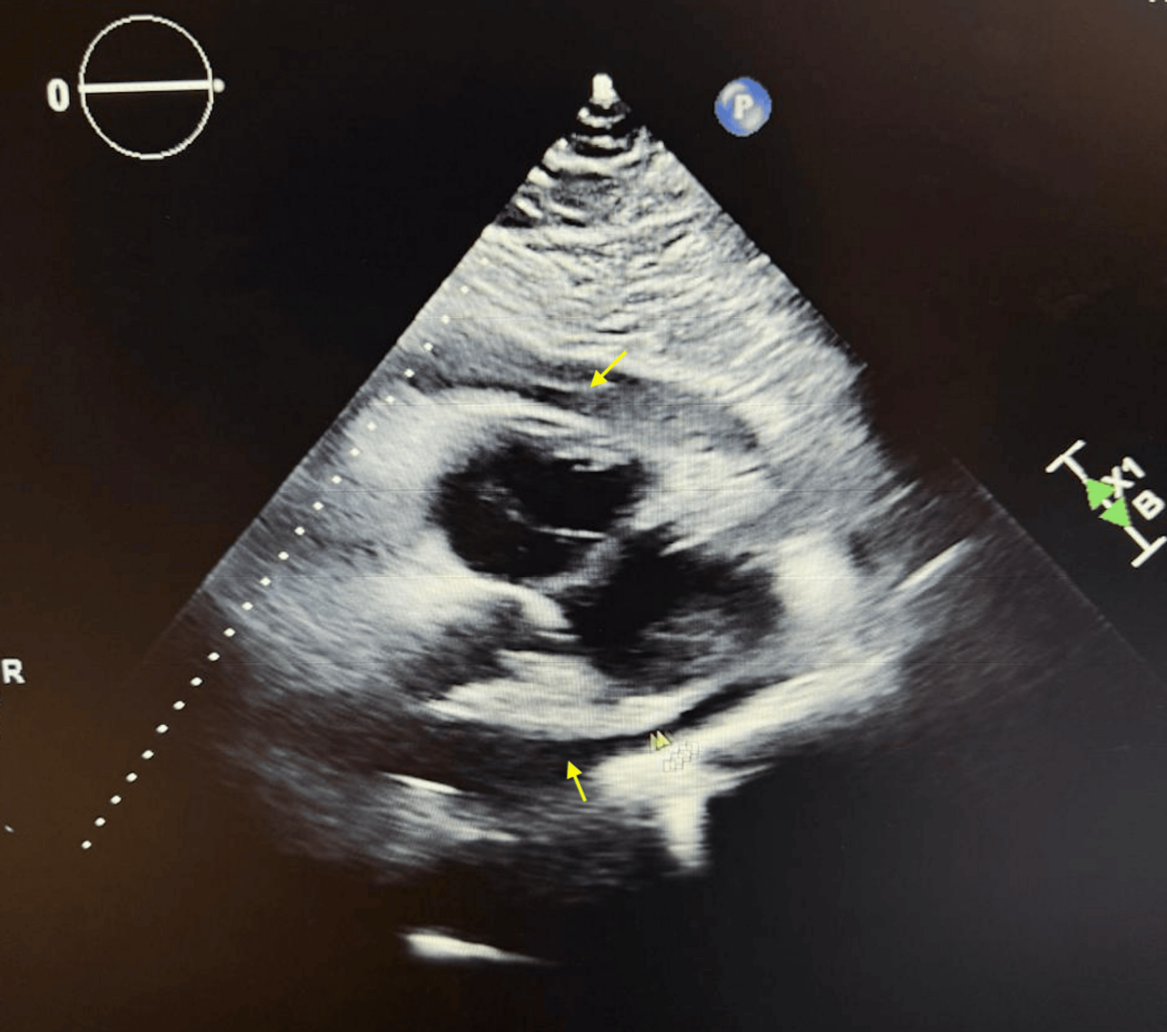
TTE (parasternal long-axis view): Circumferential pericardial effusion (yellow arrows) TTE: Transthoracic echocardiogram

Transthoracic echocardiography (TTE) revealed a preserved left ventricular ejection fraction, mild tricuspid regurgitation, and a moderate, circumferential pericardial effusion (Figure 2).

**Figure 3:**
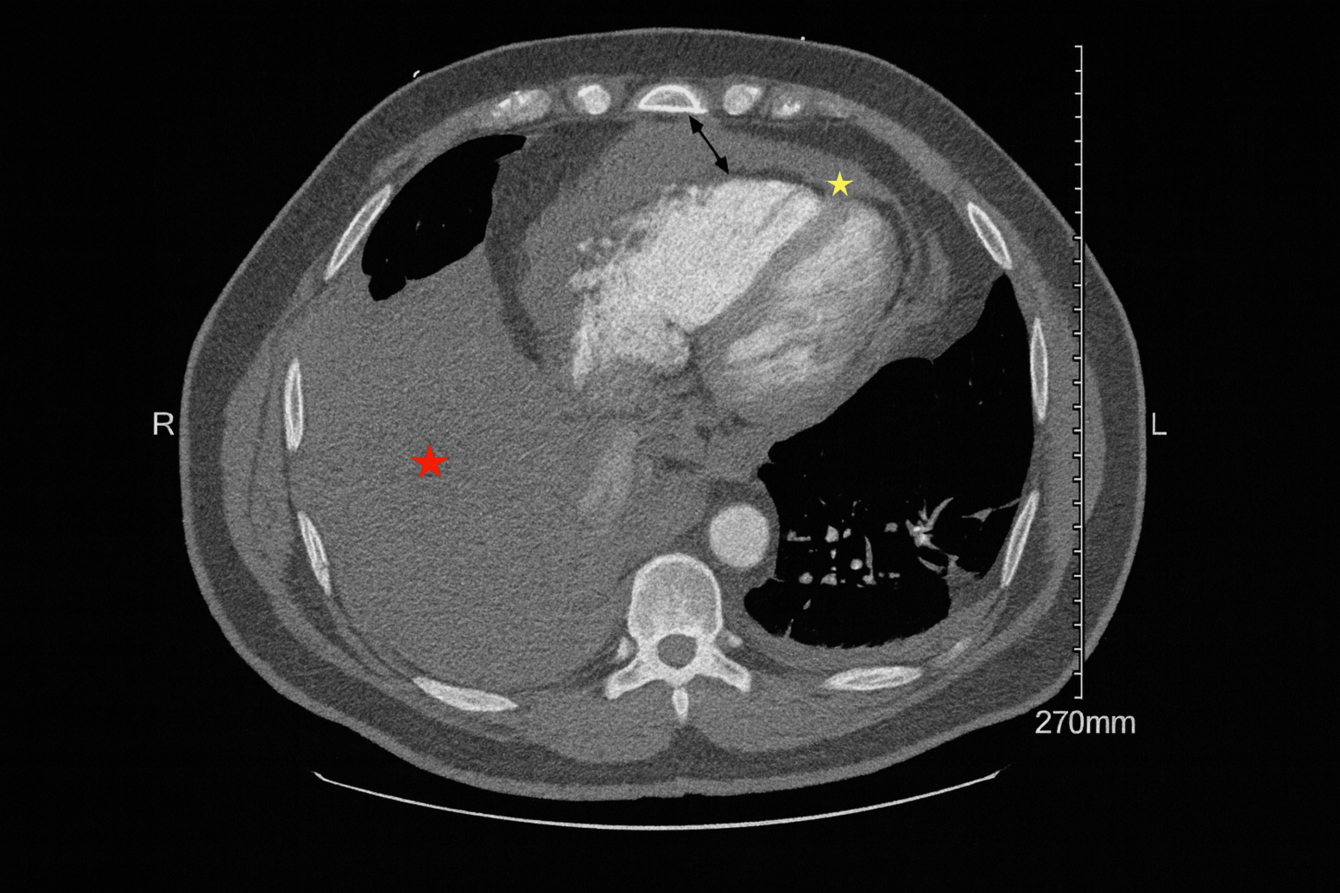
CT angiography of the chest: pericardial thickening (black arrow), moderate pericardial effusion (yellow star), and a large right pleural effusion (red star)

However, there were no overt signs of tamponade physiology; subtle echocardiographic features hinted at possible right ventricular strain. To further characterize the thoracic pathology, contrast-enhanced computed tomography (CT) angiography of the chest was performed. This demonstrated pericardial thickening with heterogeneous enhancement, a moderate pericardial effusion, bulky anterior mediastinal lymphadenopathy, a large right pleural effusion, and complete atelectasis of the right lower lobe (Figure 3).

Given the persistence and progression of symptoms despite prior interventions, the patient underwent therapeutic thoracentesis, draining 900 mL of serosanguineous fluid. Cytological and microbiological analyses again failed to reveal infectious or malignant cells. A multidisciplinary decision was made to proceed with video-assisted thoracoscopic surgery (VATS) for pleural biopsy and a subxiphoid pericardial window, which yielded an additional 500 mL of hemorrhagic fluid from the pericardial space. Postoperative anti-inflammatory therapy with colchicine and indomethacin was initiated empirically.

**Figure 4:**
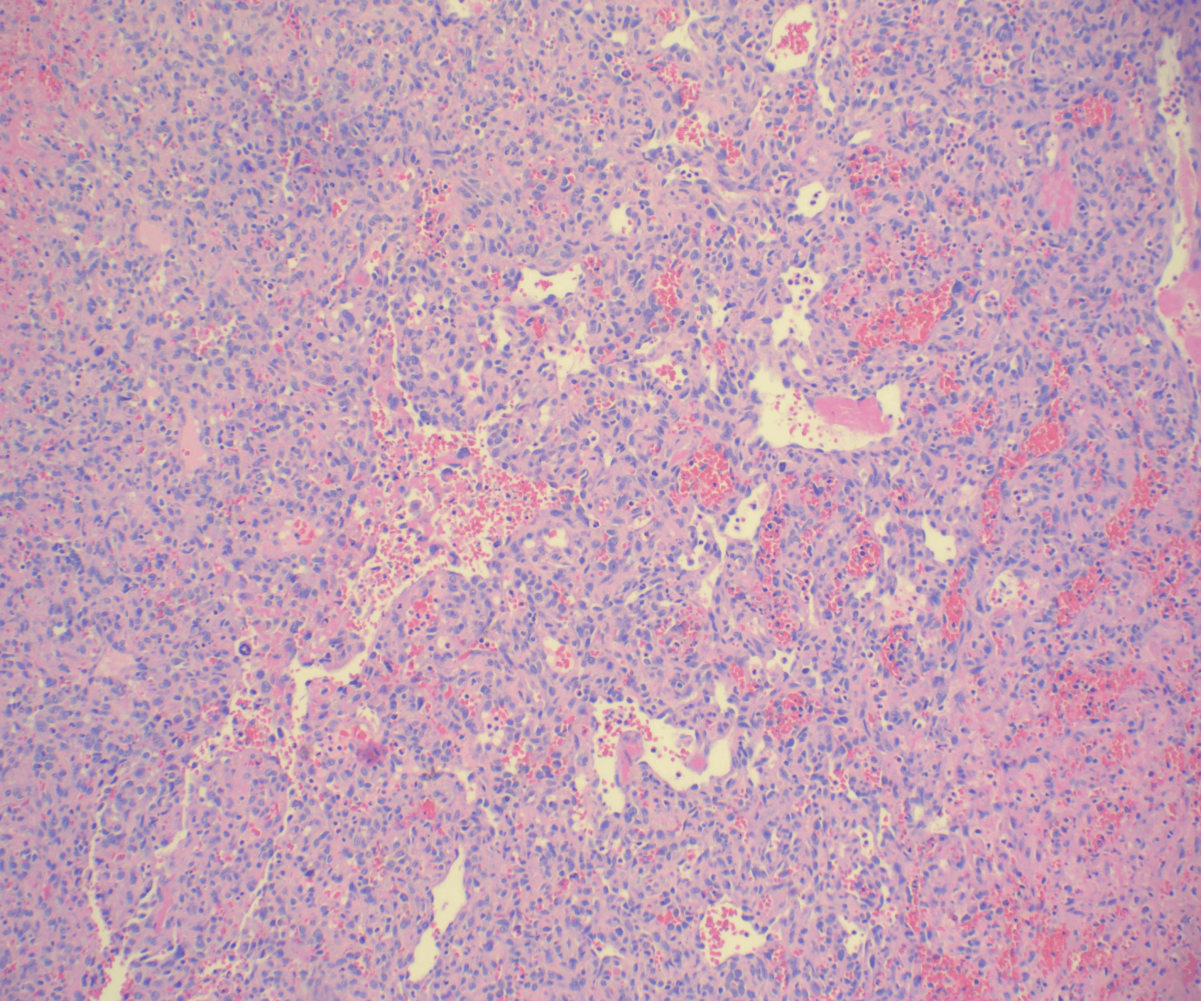
Hematoxylin and Eosin (H&E) stain pericardial biopsy at a magnification of 100, showing infiltrative sheets of epithelioid cells with prominent nucleoli and a vasoformative growth pattern

**Figure 5:**
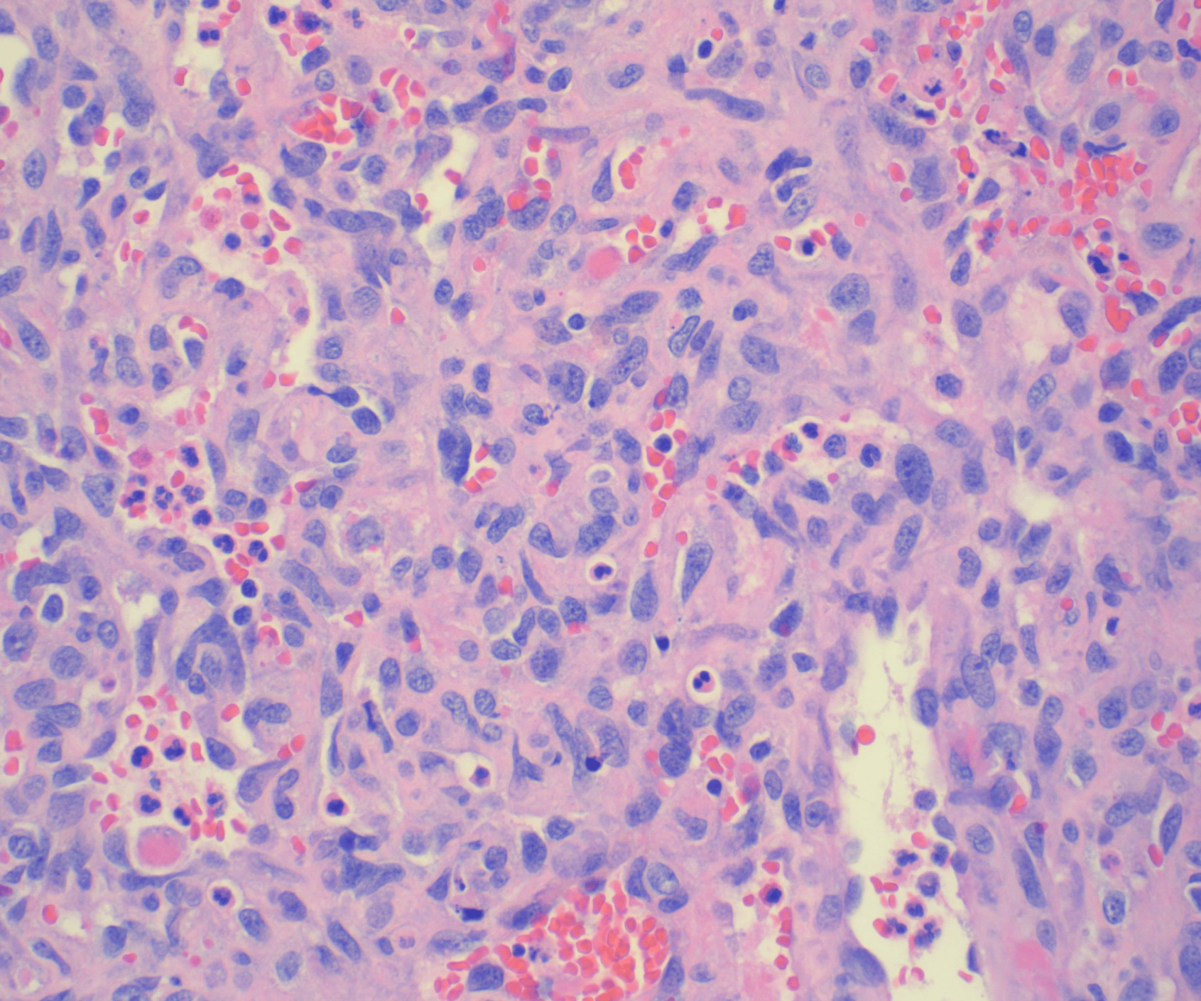
Hematoxylin and Eosin (H&E) stain pericardial biopsy at a magnification of 400, showing infiltrative sheets of epithelioid cells with prominent nucleoli and a vasoformative growth pattern

Histopathologic examination of pleural and pericardial biopsies revealed infiltrative sheets of epithelioid cells with prominent nucleoli and a vasoformative growth pattern (Figures 4-5).

**Figure 6:**
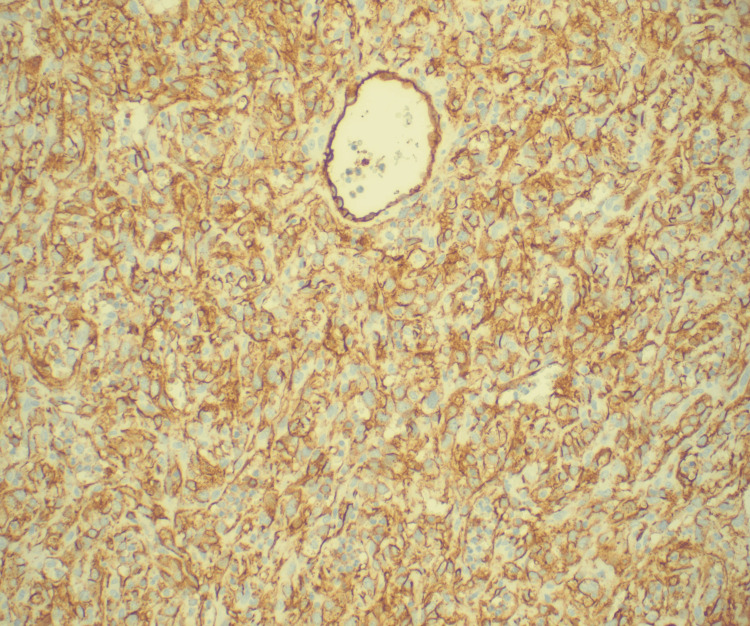
Immunohistochemical stain of the pericardial biopsy at a magnification of 200: positive for the endothelial cell marker CD31

**Figure 7:**
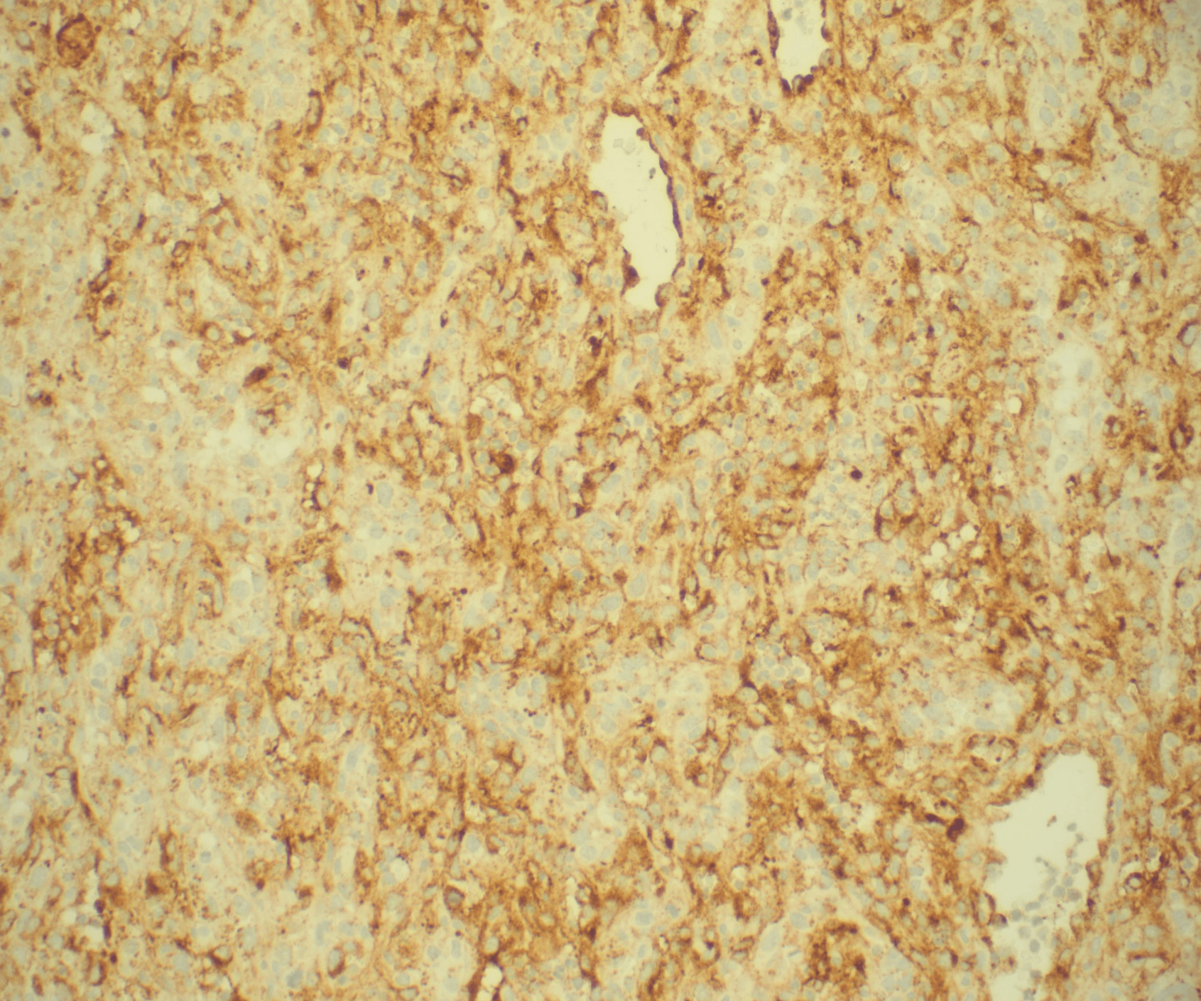
Immunohistochemical stain of the pericardial biopsy at a magnification of 200: positive for endothelial cell marker factor VIII

Immunohistochemical staining was remarkable for positivity of endothelial markers CD31, factor VIII, electroretinogram (ERG), and pan-carcinoembryonic antigen (CEA) (Figures 6-7).

Notably, tumor cells were negative for WT1, calretinin, and claudin-4, excluding mesothelial and epithelial malignancies. Retention of nuclear p40, BAP1, and MTAP further supported the diagnosis of high-grade epithelioid angiosarcoma, a rare and aggressive vascular neoplasm. A systemic metastatic workup, including positron emission tomography (PET-CT), was negative at the time of diagnosis.

## Discussion

Primary cardiac tumors are extremely rare, comprising only 0.001% to 0.28% of autopsy cases [5]. Among these, benign tumors account for the majority (75%), while malignancies represent the remaining 25% [6]. Of the malignant cardiac tumors, sarcomas are the most common, making up 90% to 95% of cases, with angiosarcomas being the most prevalent [7]. These tumors represent approximately 30% of all primary cardiac malignancies, and they most frequently arise in the right atrium, affecting up to 75% of cases [8]. Cardiac angiosarcomas predominantly impact males between the ages of 30 and 50 [9].

Due to their insidious progression and often asymptomatic nature, cardiac angiosarcomas are commonly diagnosed at advanced stages, often after metastasis has occurred. Studies show that 66% to 89% of patients experience metastasis, typically to the pericardium, particularly in cases of right-sided angiosarcomas. Other common metastatic sites include the lungs, liver, bones, and soft tissues [10]. One hallmark feature of these tumors is recurrent pericardial effusion, which may lead to tamponade. A study by Hong et al. found that 56% of patients with cardiac angiosarcomas presented with pericardial effusion, with or without tamponade. In these cases, pericardiocentesis serves as an important diagnostic tool, with cytopathological results being positive in 75% to 81% of cases [3].

For imaging, transthoracic echocardiography (TTE) is typically the first-line modality used to assess the tumor’s size, location, mobility, and attachment, offering a sensitivity of 97%. CT plays a vital role in evaluating tumor mass and detecting metastasis. However, cardiac magnetic resonance imaging (MRI) is particularly valuable for distinguishing between tumors and thrombi, providing superior imaging. On MRI, cardiac angiosarcomas typically appear isointense on T1-weighted images and hyperintense on T2-weighted images. Tumors involving the pericardium may present with a lobulated, "cauliflower-like" appearance, and contrast-enhanced MRI can reveal a characteristic "sunray" effect. Additionally, coronary angiography can help distinguish angiosarcomas from hemangiomas, as it detects tumor neovascularization, especially when the tumor arises from the right coronary artery [11].

The definitive diagnosis of cardiac angiosarcoma is made through histopathological examination and immunohistochemistry. These tumors are characterized by irregular anastomotic vessels, spindle-shaped cells, and an absence of calcification. Key immunohistochemical markers indicative of endothelial differentiation include CD31, CD34, ERG, factor VIII-related protein, von Willebrand factor, cytokeratin, vimentin, p53, Ki-67, Wilms tumor 1, alpha-smooth muscle actin, and BNH9. Among these, CD31, CD34, and factor VIII-related protein are particularly specific and confirm the diagnosis [12].

Unfortunately, the prognosis for cardiac angiosarcomas remains poor, with a median survival of only six months. Even after surgical intervention, the average postoperative survival is less than ten months. Surgical intervention is generally considered palliative rather than curative. However, studies have shown that multimodal therapy, which may include surgery, chemotherapy, radiotherapy, and even heart transplantation, can result in survival durations of up to 30 months.

Chemotherapy plays a critical role in the treatment of cardiac angiosarcoma, although its effectiveness is limited. Common chemotherapeutic agents include doxorubicin and ifosfamide, both of which can reduce tumor mass and potentially make radical surgery more feasible. Other chemotherapy agents, such as paclitaxel, docetaxel, gemcitabine, cyclophosphamide, vincristine, and dacarbazine, have been used with varying degrees of success. Additionally, case reports suggest that angiosarcomas may respond to targeted therapies such as pazopanib, imatinib, recombinant interleukin-2, and bevacizumab [12].

In this case, the patient’s presentation with recurrent pleural and pericardial effusions, despite the absence of a right atrial mass on TTE, was atypical for more common causes. CT imaging revealed pericardial involvement and mediastinal lymphadenopathy, prompting further investigation. The diagnosis of cardiac angiosarcoma was ultimately confirmed through immunohistochemical staining for ERG, a key marker for angiosarcoma [6]. This case emphasizes the importance of a comprehensive diagnostic approach, especially in patients presenting with recurrent effusions or other nonspecific symptoms, when more common conditions have been ruled out. A multidisciplinary approach, including advanced imaging and histopathological examination, is critical to establishing an accurate diagnosis and guiding appropriate management.

## Conclusions

Cardiac angiosarcomas, though exceedingly rare, present significant diagnostic and treatment challenges due to their often-subtle presentation, aggressive nature, and propensity for metastasis. While imaging and histopathology are critical in achieving a diagnosis, the prognosis remains poor, with treatment options generally limited to palliative care. However, advances in multimodal therapy and targeted treatments offer hope for extending survival in select patients, even if curative outcomes remain elusive.

## References

[1] Suehiro Y, Kajiyama T, Satoh A (2023). Successful surgical treatment for primary cardiac angiosarcoma: A case report. Gen Thorac Cardiovasc Surg Cases.

[2] Kumari N, Bhandari S, Ishfaq A (2023). Primary cardiac angiosarcoma: A review. Cureus.

[3] Patel SD, Peterson A, Bartczak A, Lee S, Chojnowski S, Gajewski P, Loukas M (2014). Primary cardiac angiosarcoma - a review. Med Sci Monit.

[4] Luo L, Zhao W, Liu K (2021). Cardiac Angiosarcoma: A case report and review of the literature. Authorea.

[5] Burke A, Tavora F (2016). The 2015 WHO classification of tumors of the heart and pericardium. J Thorac Oncol.

[6] Patel J, Sheppard MN (2010). Pathological study of primary cardiac and pericardial tumours in a specialist UK Centre: Surgical and autopsy series. Cardiovasc Pathol.

[7] Bakaeen FG, Jaroszewski DE, Rice DC (2009). Outcomes after surgical resection of cardiac sarcoma in the multimodality treatment era. J Thorac Cardiovasc Surg.

[8] Oliveira GH, Al-Kindi SG, Hoimes C, Park SJ (2015). Characteristics and survival of malignant cardiac tumors: A 40-year analysis of >500 patients. Circulation.

[9] Hoffmeier A, Sindermann JR, Scheld HH, Martens S (2014). Cardiac tumors—diagnosis and surgical treatment. Dtsch Arztebl Int.

[10] Krishnan T, Pettersson G, Mukherjee R, Singhal N (2020). Cardiac angiosarcoma: A diagnostic and therapeutic challenge. J Cardiol Cases.

[11] Thiene G (2014). Sudden cardiac death and cardiovascular pathology: From anatomic theater to double helix. Am J Cardiol.

[12] Shanmugam G (2006). Primary cardiac sarcoma. Eur J Cardiothorac Surg.

